# Down-regulation of the zinc-finger homeobox protein TSHZ2 releases GLI1 from the nuclear repressor complex to restore its transcriptional activity during mammary tumorigenesis

**DOI:** 10.18632/oncotarget.6788

**Published:** 2015-12-29

**Authors:** Miho Riku, Shingo Inaguma, Hideaki Ito, Takumi Tsunoda, Hiroshi Ikeda, Kenji Kasai

**Affiliations:** ^1^ Department of Pathology, Aichi Medical University School of Medicine, Nagakute, Aichi, Japan

**Keywords:** TSHZ2, GLI1, CXCR4, AEBP1, breast cancer

## Abstract

Although breast cancer is one of the most common malignancies, the molecular mechanisms underlying its development and progression are not fully understood. To identify key molecules involved, we screened publicly available microarray datasets for genes differentially expressed between breast cancers and normal mammary glands. We found that three of the genes predicted in this analysis were differentially expressed among human mammary tissues and cell lines. Of these genes, we focused on the role of the zinc-finger homeobox protein TSHZ2, which is down-regulated in breast cancer cells. We found that TSHZ2 is a nuclear protein harboring a bipartite nuclear localization signal, and we confirmed its function as a C-terminal binding protein (CtBP)-dependent transcriptional repressor. Through comprehensive screening, we identified TSHZ2-suppressing genes such as *AEBP1* and *CXCR4*, which are conversely up-regulated by GLI1, the downstream transcription factor of Hedgehog signaling. We found that GLI1 forms a ternary complex with CtBP2 in the presence of TSHZ2 and that the transcriptional activity of GLI1 is suppressed by TSHZ2 in a CtBP-dependent manner. Indeed, knockdown of TSHZ2 increases the expression of AEBP1 and CXCR4 in TSHZ2-expressing immortalized mammary duct epithelium. Concordantly, immunohistochemical staining of mammary glands revealed that normal duct cells expresses GLI1 in the nucleus along with TSHZ2 and CtBP2, whereas invasive ductal carcinoma cells, which does not express TSHZ2, show the increase in the expression of AEBP1 and CXCR4 and in the cytoplasmic localization of GLI1. Thus, we propose that down-regulation of TSHZ2 is crucial for mammary tumorigenesis *via* the activation of GLI1.

## INTRODUCTION

Breast cancer is one of the most common malignancies and is increasing fatal worldwide. To date, hundreds of gene expression analyses of breast cancer cells have been performed that identified distinct cellular subtypes and molecular differences among breast cancer patients. However, the molecular changes during mammary tumorigenesis have not been fully elucidated.

Hedgehog (Hh) signaling is key for cell fate determination and tissue patterning during embryonic development and adult tissue restoration. Malfunction of this signaling pathway causes numerous developmental anomalies and in turn its dysregulated activation impacts on the development and progression of many human malignancies, including breast cancer [1, 2]. Indeed, the expression of GLI1, a key transcription factor in the downstream of Hh signaling, clinically associates with unfavorable prognosis of breast cancer patients [3], and experimentally the conditional expression of GLI1 in a transgenic mouse model induced mammary tumors [4], suggesting a crucial role of GLI1 in breast cancer development.

In the canonical pathway of Hh signaling, Hh secreted glycoprotein (Sonic, Indian and Desert hedgehog) associates with a receptor Patched (Ptch) to derepress a seven-transmembrane signal transducer Smoothened (SMO), leading to the expression of *GLI1* through a heterotrimeric G-protein-dependent or independent cascades [1]. In addition, the non-canonical pathway of Hh signaling plays a crucial role for the activation of *GLI1* expression, especially in the development and progression of cancer: transforming growth factor-β (TGFβ) pathway induces SMAD3-dependent transcriptional activation of *GLI2* gene and the consequent GLI2-induced expression of *GLI1* [5]. However, a key step for a full activation of Hh/GLI1 signaling is to enhance the nuclear accumulation and transcriptional activity of GLI1: GLI1 is predominantly expressed in the cytoplasm of cells, but is well-known to shuttle between the cytoplasm and nucleus [6]. And either canonical or non-canonical pathway of Hh signaling somehow enhance the nuclear accumulation and transcriptional activity of GLI1: we reported that oncogenic mutation of *K-RAS* and the activated downstream MAPK/ERK pathway are involved in the derepression of GLI1 from the negative control of Suppressor-of-Fused (SUFU) to enhance the nuclear accumulation and transcriptional activity of GLI1 in pancreatic ductal adenocarcinoma cells [7]. However, in contrast to pancreatic duct epithelium that does not express GLI1, normal duct epithelium of the mammary glands was reported to express GLI1 predominantly in the nucleus, whereas the expression of GLI target gene was not detected [3]. Thus, the mechanisms by which normal duct epithelium suppresses the transcriptional activity of nuclear GLI1 and by which breast cancer cells activate it remain unclear.

In the present study, through the analysis and validation of publicly available microarray datasets, we identified *TSHZ2*, a zinc-finger homeobox gene, as one of three genes down-regulated in breast cancer cells compared with normal mammary glands. We found that TSHZ2 is a nuclear protein that acts as a transcriptional repressor in the presence of the CtBP corepressor protein. Comprehensive screening of genes down-regulated by TSHZ2 revealed that some, but not all, of these genes were up-regulated by GLI1 in a breast cancer cell line. We found that GLI1 formed a ternary complex with CtBP2 in the presence of TSHZ2. Concordantly, knockdown of TSHZ2 in TSHZ2-expressing immortalized mammary duct epithelium revealed the increased expression of GLI1 target genes, including the adipocyte enhancer-binding protein AEBP1 and the chemokine receptor CXCR4. Furthermore, through immunohistochemical analysis, we found that normal duct epithelium expressed GLI1 predominantly in the nucleus along with TSHZ2 and CtBP2: breast cancer cells, which did not express TSHZ2 but CtBP2, exhibited the increase in the expression of AEBP1 and CXCR4 and also in the cytoplasmic distribution of GLI1. Thus, we propose that the down-regulation of TSHZ2 is a key step toward activating pre-existing GLI1 in mammary duct epithelium, leading to increased activity of the Hh/GLI1 signaling cascade.

## RESULTS

### TSHZ2 is down-regulated in breast cancer

**Figure 1:**
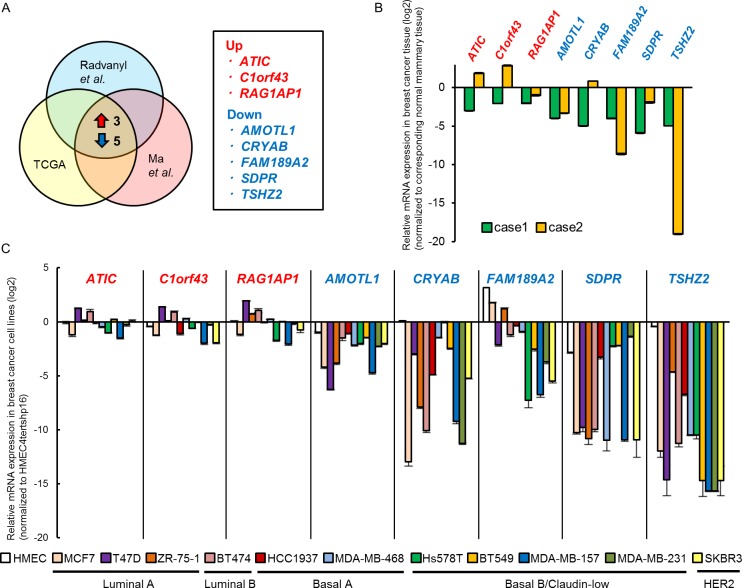
TSHZ2 is down-regulated in breast cancer **A.** Schema of the identification of candidate genes involved in mammary tumorigenesis. The top 5% of genes differentially expressed between normal mammary glands and breast cancers were compared and three up-regulated (*ATIC*, *C1orf43*, *RAG1AP1*) and five down-regulated (*AMOTL1*, *CRYAB*, *FAM189A2, SDPR* and *TSHZ2*) genes were shared among datasets (see also Supplementary Figure S1). **B.** qRT-PCR analysis of gene expression in breast cancer tissue and corresponding normal mammary tissue. Data are presented relative (log2 scale) to the expression level in corresponding normal mammary tissue. *Columns*, mean values; *bars*, SD. **C.** qRT-PCR analysis of gene expression in human breast cancer cell lines as well as normal mammary epithelium in primary culture (HMEC) and immortalized normal mammary epithelium (HMEC4*htertshp16*). Data are presented relative (log2 scale) to the expression level in HMEC4*htertshp16* cells. *Columns*, mean values; *bars*, SD.

To identify candidate genes involved during mammary tumorigenesis, we examined the following seven independent microarray datasets: “Ductal carcinoma *in situ* (DCIS) epithelium *versus* normal breast epithelium” and “invasive ductal breast carcinoma *versus* normal breast epithelium” (GSE14548) [8]; “DCIS *versus* normal tissue”, “invasive ductal breast carcinoma *versus* normal tissue” and “invasive lobular breast carcinoma *versus* normal tissue” (GSE1477) [9]; and “invasive ductal breast carcinoma *versus* normal tissue” and “invasive breast carcinoma *versus* normal tissue” (TCGA). From each dataset, we selected the top 5% of genes that were differentially expressed between breast cancer cells and normal breast epithelium, and then extracted three up-regulated (*ATIC*, *C1orf43*, *RAG1AP1*) and five down-regulated genes (*AMOTL1*, *CRYAB*, *FAM189A2, SDPR* and *TSHZ2*) shared among the seven datasets (Figure 1A; see Supplementary Figure S1 for details). To validate the differential expression of the genes, we employed qRT-PCR analysis of the following samples: as for human mammary glands, we used RNA samples extracted from breast cancer tissues of two independent cases and their corresponding normal mammary tissues as a control (Figure 1B); as for human cultured cells, we used RNA samples extracted from eleven human breast cancer cell lines of distinct intrinsic subtypes [10] and primary culture of normal mammary epithelium (HMEC), and immortalized normal mammary epithelium (HMEC4*htertshp16*) as a control (Figure 1C). We found that of these eight genes only three (*AMOTL1*, *SDPR* and *TSHZ2*) exhibited the expression pattern predicted by the microarray datasets (Figure 1B and 1C): namely, these three genes were down-regulated in both human breast cancer tissues (compared to normal mammary tissue; Figure 1B) and breast cancer cell lines (compared to HMEC4*htertshp16*; Figure 1C).

Among these three genes, the roles of AMOTL1 and SDPR in cancer cell biology were largely analyzed: AMOTL1 (angiomotin-like 1) and its paralog ANGIOMOTIN (AMOT) suppresses the Hippo pathway by interacting with the YAP and TAZ effectors [11]; the down-regulation of *SDPR/CAVIN2* mRNA affects caveola formation in breast cancer cells [12]. The down-regulation of *TSHZ2* mRNA was also reported in breast and prostate cancer cell lines [13]. However, unlike *AMOTL1* and *SDPR*, the role of TSHZ2 down-regulation in cancer cells has not been fully elucidated. We therefore focused our analysis on TSHZ2.

### TSHZ2 is a *bona fide* nuclear protein

**Figure 2:**
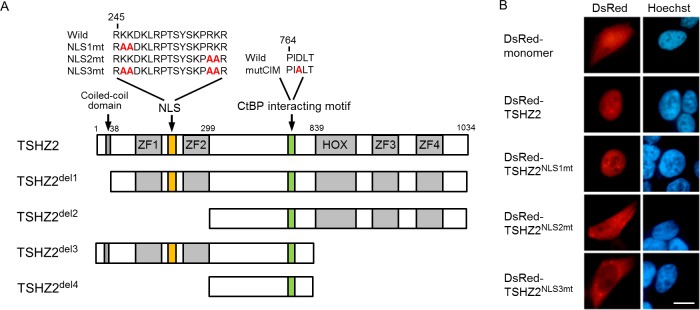
TSHZ2 is a nuclear protein **A.** Structure of human TSHZ2 as well as mutated and deleted constructs. **B.** Subcellular localization of DsRed-TSHZ2 and mutant proteins in transiently transfected HEK293T cells. *Bar*, 10 μm.

TSHZ2, a mammalian homolog of *Drosophila* Teashirt/Tiptop, contains four zinc-finger (ZF) domains, a homeobox domain and a coiled-coil domain (Figure 2A) [14]. However, the region responsible for the subcellular localization of TSHZ2 has not been identified. We therefore examined the subcellular localization of TSHZ2 by transfecting DsRed-tagged expression vectors into HEK293T cells. We observed the nuclear accumulation of DsRed-tagged TSHZ2 (DsRed-TSHZ2) (Figure 2B). Consistently, nuclear localization signal (NLS) prediction software (cNLS Mapper) [15] indeed indicated a bipartite nuclear localization signal (245-RKKDKLRPTSYSKPRKR) between the first and second ZF domains (Figure 2A). Thus, we constructed the expression vectors for DsRed-TSHZ2 harboring a mutated NLS (NLS1mt, 2mt and 3mt) and examined their subcellular localization by transfection. We found that DsRed-TSHZ2^NLS3mt^, but neither DsRed-TSHZ2^NLS1mt^ nor DsRed-TSHZ2^NLS2mt^, was excluded from the nucleus (Figure 2B; Figure 2A for mutated NLS sequence), indicating responsibility for the nuclear accumulation. Furthermore, immunohistochemical analysis revealed that endogenous TSHZ2 protein was expressed in the nuclei of normal duct epithelium and myoepithelium of human mammary glands as shown below (see Figure 6A for immunohistochemical staining). These evidences indicated that TSHZ2 is a *bona fide* nuclear protein.

### TSHZ2 functions as a CtBP-dependent transcription repressor

**Figure 3:**
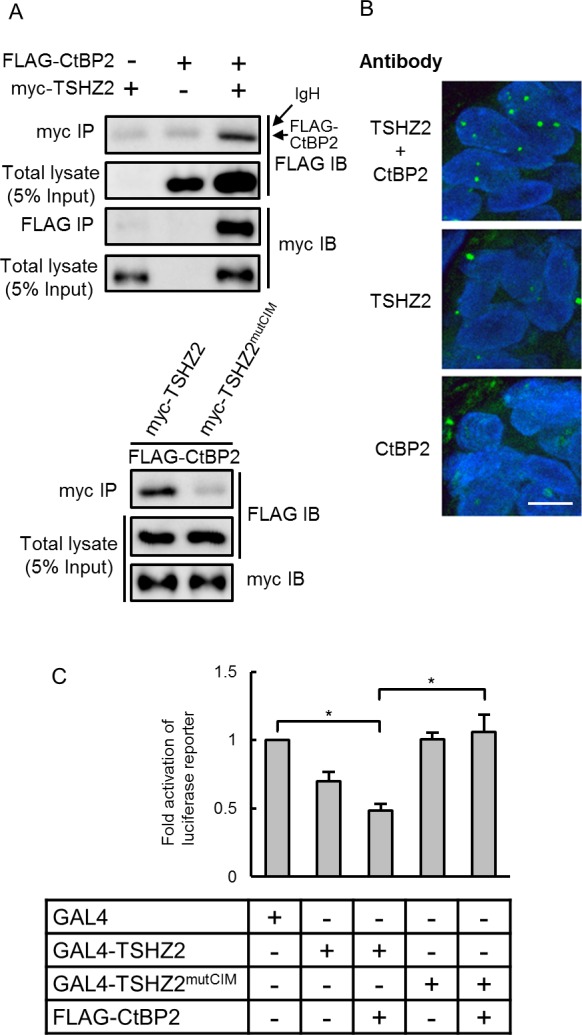
TSHZ2 is a CtBP-dependent transcriptional repressor **A.** Immunoprecipitation analysis. HEK293T cells were transiently transfected with the indicated expression vectors and used in immunoprecipitation analysis. IgH indicates the immunoglobulin heavy chain of the anti-myc tag antibody weakly cross-reacted to a secondary antibody for immunodetection. **B.** *In situ* PLA was performed using tissue sections cut from human mammary tissue blocks. DAPI was used to mark nuclei. *Bar*, 10 μm. **C.** Luciferase reporter assay. Cells were transiently transfected with either GAL4 or GAL4-fused TSHZ2 expression vector (wild-type or mutCIM) along with pG5TKluc, UAS-containing thymidine kinase minimal promoter-driven luciferase vector, and *Renilla* luciferase vector. *Columns*, means of three independent experiments; *bars*, SD. *P < 0.01.

*Drosophila* Teashirt/Tiptop was previously reported to harbor an interaction motif for the transcriptional corepressor CtBP (CtBP-interaction motif; *hereafter*, CIM) [14]. We also found a putative CIM (764-PIDLT) in human TSHZ2 (Figure 2A). Indeed, immunoprecipitation analysis revealed that myc-tagged TSHZ2, but not TSHZ2 harboring a mutated CIM (764-PIALT; mutCIM, Figure 2A), associated with FLAG-tagged CtBP2 in HEK293T cells (Figure 3A). Unfortunately, we failed to immunoprecipitate either endogenous TSHZ2 or CtBP1/2 proteins efficiently from *TSHZ2*-expressing HMEC4*htertshp16* cells due to the poor accessibility of antibodies to these proteins that might be incorporated in a multi-molecular repressor complex (data not shown). However, *in situ* proximity ligation assay (PLA) using 2 μm human mammary tissue sections revealed that endogenous TSHZ2 associated with endogenous CtBP2 in the nucleus of normal human mammary gland duct epithelium (Figure 3B), suggesting their association *in vivo*. Furthermore, we found that TSHZ2 suppressed the thymidine kinase minimal promoter (TKmini)-driven activation of the luciferase reporter gene in the CIM-dependent manner (Figure 3C). Taken together with the nuclear expression of TSHZ2 and CtBP2 in normal duct epithelium as shown below (Figure 6A), these data indicate that TSHZ2 is a nuclear protein that acts as a CtBP-dependent transcriptional repressor in normal duct epithelium.

### TSHZ2 associates with and accumulates GLI1 in nucleus

**Figure 4:**
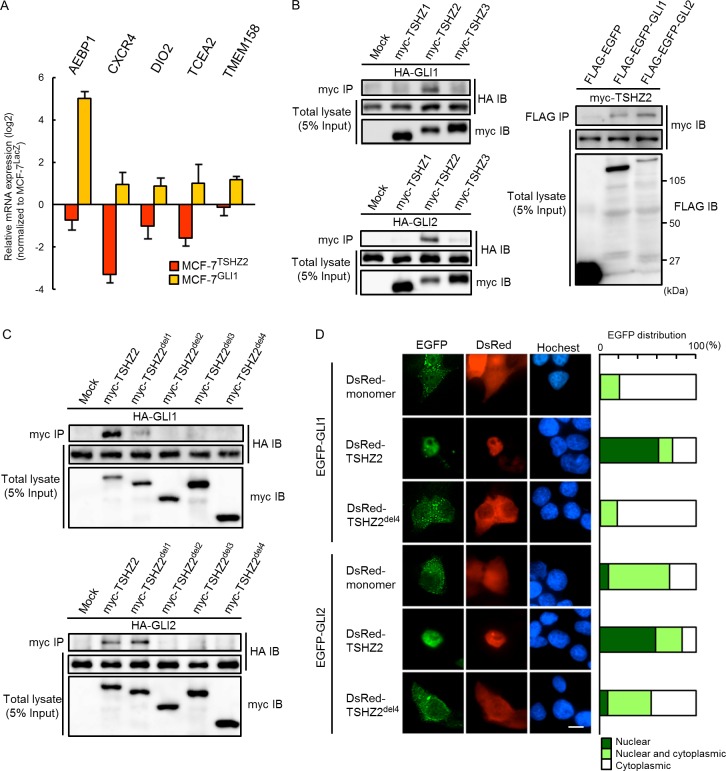
TSHZ2 associates with and accumulates GLI1 in nucleus **A.** qRT-PCR analysis of MCF-7^TSHZ2^, MCF-7^GLI1^ and MCF-7^LacZ^ gene expression. Data are presented relative (log2 scale) to MCF-7^LacZ^. *Columns*, mean values; *bars*, SD. **B.** and **C.** Immunoprecipitation analysis. HEK293T cells were transiently transfected with the indicated expression vectors and used in immunoprecipitation analysis. **D.** Subcellular localization of EGFP-GLI1 and EGFP-GLI2 in transiently transfected HEK293T cells. *Right*, The ratio of the subcellular distribution of EGFP or EGFP-fused proteins. The result was based on the estimation of more than one hundred transfectants. Bar, 10μm.

Given that TSHZ2 acts as a transcriptional repressor and is down-regulated in breast cancer cells (Figure 1B and 1C; see Figure 6A for immunohistochemical staining), we investigated genes up-regulated in breast cancer cells upon TSHZ2 down-regulation. To this end, we established either TSHZ2 or a control LacZ-expressing cells from a human breast cancer cell line MCF-7 (MCF-7^TSHZ2^ and MCF-7^LacZ^, respectively) and searched for genes differentially expressed between MCF-7^TSHZ2^ and MCF-7^LacZ^ *via* expression microarray analysis. We identified 1,612 up-regulated and 1,114 down-regulated genes dependent on TSHZ2 expression (GSE64351). Intriguingly, *AEBP1*, *CXCR4*, *DIO2* (*deiodinase, iodothyronine, typeII*), *TCEA2* (*transcription elongation factor SII, 2*) and *TMEM158* (*transmembrane protein 158*) were among the down-regulated genes in MCF-7^TSHZ2^ cells, whereas we previously identified these as genes up-regulated in GLI1-overexpressing MCF-7^GLI1^ cells (GSE64350; Figure 4A) [16]. We therefore sought to understand whether TSHZ2 interfered with the transcriptional activity of GLI1. We first examined the association of GLI1 and GLI2 with TSHZ2, as well as TSHZ1 and TSHZ3 (the other members of the TSHZ protein family), by immunoprecipitation assay. We found that both HA-tagged GLI1 and GLI2 co-immunoprecipitated with myc-TSHZ2 but not myc-TSHZ1 or myc-TSHZ3, and that myc-TSHZ2 reciprocally co-immunoprecipitated with FLAG-EGFP-tagged GLI1 and GLI2 (Figure 4B). Using deletion variants of TSHZ2, we found that both the ZF1-2 and the Hox/ZF3-4 domains were required for the association with GLI1 and GLI2 (Figure 4C; see Figure 2A for deletion constructs). GLI1 shuttles between the cytoplasm and nucleus but is well-known to be observed predominantly in the cytoplasm [6], as shown in Figure 4D. However, we found that in the presence of DsRed-TSHZ2 but not DsRed-TSHZ2^del4^, EGFP-tagged GLI1 and GLI2 accumulated predominantly in the nuclei of HEK293T cells (Figure 4D). This evidence is consistent with the predominant localization of endogenous GLI1 in the nucleus of normal duct epithelium in contrast to the cytoplasmic distribution or both the nucleus and cytoplasm distribution of GLI1 in invasive ductal carcinoma of no special type (IDC) cells as shown below (see Figure 6A for immunohistochemical staining).

### TSHZ2 suppresses transcription activity of GLI1

**Figure 5:**
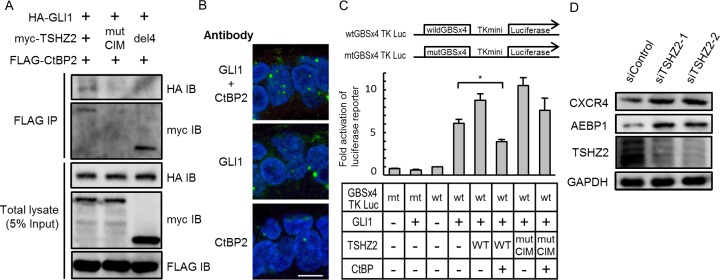
TSHZ2 suppresses the transcriptional activity of GLI1 **A.** Immunoprecipitation analysis. HEK293T cells were transiently transfected with the indicated expression vectors and used in immunoprecipitation analysis. **B.** *In situ* PLA was performed using tissue sections cut from human mammary tissue blocks. DAPI was used to mark nuclei. *Bar*, 10 μm. **C.** Luciferase reporter assay. Cells were transiently transfected with the indicated expression vectors and either wtGBSx4TKLuc or mtGBSx4TKLuc. To normalize the transfection efficiency, *Renilla* luciferase vector was also transfected. *Columns*, mean of three independent experiments; *bars*, SD. *P < 0.01. **D.** Immunoblot analysis of siRNA-transfected HMEC4*htertshp16* cells.

We next ascertained whether TSHZ2 suppressed the transcriptional activity of GLI1. Immunoprecipitation analysis revealed that HA-GLI1 co-immunoprecipitated with FLAG-CtBP2 in the presence of myc-TSHZ2, and that the association between HA-GLI1 and FLAG-CtBP2 was dependent on the CIM of TSHZ2 (Figure 5A; see Supplementary Figure S2 for HA-GLI2 immunoprecipitation). Furthermore, *in situ* PLA revealed that endogenous GLI1 associated with endogenous CtBP2 in the nucleus of normal human mammary gland duct epithelium (Figure 5B). These results indicated that GLI1 forms a ternary complex with CtBP2 in the presence of TSHZ2. Concordantly, reporter assay using GLI-binding site-containing luciferase reporter constructs revealed that TSHZ2 suppressed the transcriptional activity of GLI1 in a CtBP2-dependent manner (Figure 5C). In addition, we found that the expression of AEBP1 and CXCR4, which are direct target genes of GLI1 [16] (Supplementary Figure S3 for the direct *AEBP1* regulation by GLI1), was increased following knockdown of TSHZ2 in HMEC4*htertshp16* cells (Figure 5D). Furthermore, being consistent with TSHZ2-mediated suppression of CXCR4 expression (Figure 4A), we found that CXCL12-induced breast cancer cell migration was inhibited by the expression of TSHZ2 but neither LacZ nor TSHZ2^del4^ (Supplementary Figure S4). Taken together, these evidences indicated that in normal mammary gland duct epithelium TSHZ2 binds GLI1 to a CtBP-containing transcriptional repressor complex, leading the supression of GLI1-target gene expression.

### Expression of TSHZ2, GLI1 and targets in mammary glands

**Figure 6:**
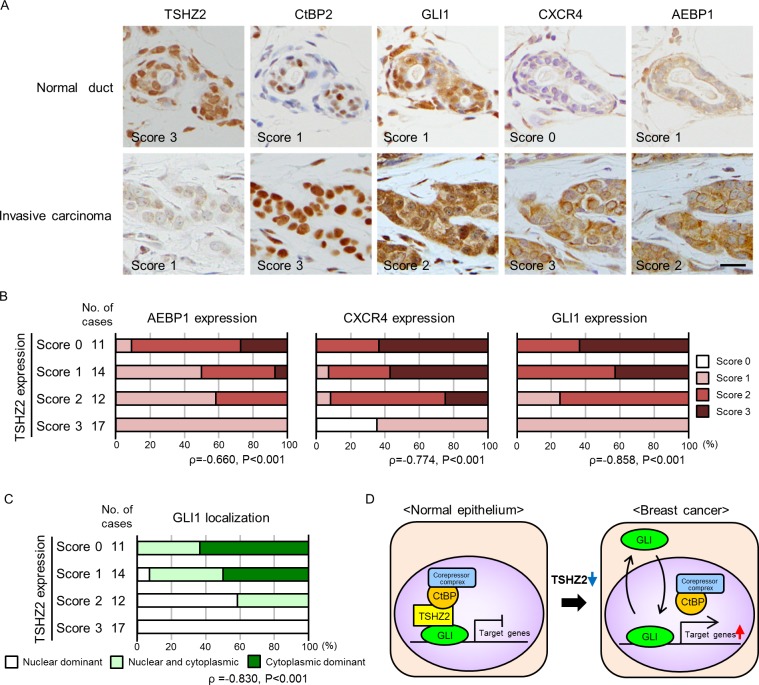
Immunohisctochemical analysis of human mammary glands **A.** Immunohistochemical staining of human mammary tissues. The expression of TSHZ2, CtBP2, GLI1, CXCR4 and AEBP1 was evaluated by staining intensity and semi-quantitatively scored (negative, 0; weak, 1; moderate, 2; strong, 3) as indicated in the photos. *Bar*, 20 μm. **B.** Correlation of TSHZ2 expression with AEBP1, CXCR4 and GLI1 expression. **C.** Correlation of TSHZ2 expression with the subcellular distribution of GLI1. **D.** A schematic of the proposed mechanism for GLI1 activation during mammary tumorigenesis. In normal mammary gland duct epithelium, GLI1 is sequestered in nucleus and its transcriptional activity is suppressed by the TSHZ2-CtBP2 repressor complex. During tumorigenesis, TSHZ2 is down-regulated, and in turn, GLI1 is released from the TSHZ2-containing repressor complex and is shuttled between subcellular compartments, thereby enabling the transactivation of target gene expression.

Finally, we examined the expression of TSHZ2, CtBP2, GLI1, CXCR4 and AEBP1 in human mammary glands. We selected 36 cases of invasive ductal carcinoma of no special type (IDC) (20 cases of estrogen receptor (ER)-positive cancer and 16 cases of ER-negative cancer) along with 18 normal tissues from the archives of the Department of Pathology at Aichi Medical University Hospital, based on the availability, and served them for immunohistochemical analysis. The immunoreactivity was semiquantitatively scored with a three-tiered scale. In accordance with the qRT-PCR analysis of breast cancer tissues and cell lines (Figure 1B and 1C), we found that TSHZ2 was strongly expressed in the nuclei of normal duct epithelium, as well as myoepithelium of human mammary glands, whereas its expression level was markedly reduced in IDC cells (Figure 6A for representative photos; Table for the statistical analysis; Supplementary Table for the details). CtBP2 was expressed in the nuclei of normal duct epithelium and IDC cells (Figure 6A, Table 1). Immunofluorescence staining demonstrated that TSHZ2 and CtBP2 were co-localized in the nuclei of normal duct epithelium (Supplementary Figure S5). We also recognized that the expression level of CtBP2 was higher in IDC cells than normal duct epithelium, supposedly due to the possibility that GLI1 enhances the transcription of CtBP2, as previously reported [17]. And we found that normal duct epithelium expressed GLI1 predominantly in nucleus along with TSHZ2 and CtBP2, whereas IDC cells showed GLI1 expression mainly in the cytoplasm or both nucleus and cytoplasm with the increased expression level of GLI1 (Figure 6A, Table 1). As for AEBP1 and CXCR4, we found that the expression levels of these GLI1 target gene products were statistically increased in IDC cells (Figure 6A, Table 1). Next, we compared TSHZ2 expression level with AEBP1, CXCR4 and GLI1 expression level and also with the subcellular distribution of GLI1. We found that the expression level of TSHZ2 was statistically related inversely with the expression level of AEBP1 and CXCR4 as well as GLI1 (Figure 6B). And we also found that the reduction of TSHZ2 expression level statistically correlated with the dominance of cytoplasmic distribution of GLI1 (Figure 6C). Based on a previous report showing that GLI1 up-regulates the expression of *GLI1* mRNA [18], these analyses suggested that the reduction of TSHZ2 expression in IDC cells associates with the increase in the transcriptional activity of GLI1, leading to the up-regulation of GLI1 target genes such as AEBP1, CXCR4 as well as GLI1 itself.

**Table 1:** The protein expression and GLI1 subcellular localization in normal mammary duct epithelium and invasive ductal carcinoma cells

	Normal	Invasive carcinoma			
		Total cases	ER (+) cases	ER (-) cases	*p*-value^a^
**Intensity (mean±SD)**					
TSHZ2	2.94 (±0.24)	1.00 (±0.79)	1.55 (±0.51)	0.31 (±0.48)	<0.05^b^
CtBP2	1.06 (±0.24)	2.00 (±0.79)	1.85 (±0.81)	2.19 (±0.75)	<0.05^b^
GLI1	1.06 (±0.24)	2.31 (±0.58)	2.05 (±0.51)	2.63 (±0.50)	<0.05^b^
CXCR4	0.67 (±0.49)	2.47 (±0.56)	2.35 (±0.59)	2.63 (±0.50)	<0.05^b^
AEBP1	1.06 (±0.24)	1.72 (±0.66)	1.45 (±0.51)	2.06 (±0.68)	<0.05^b^
**GLI1 localization (n, %)**					
Nuclear dominant	16 (89%)	7 (19%)	7 (35%)	0 (0%)	
Nuclear & cytoplasmic	2 (11%)	15 (42%)	10 (50%)	5 (31%)	<0.05^c^
Cytoplasmic dominant	0 (0%)	14 (39%)	3 (15%)	11 (69%)	

a Normal vs Total cases of invasive carcinoma;

b Mann-Whitney U-test;

c chi-square test

## DISCUSSION

Dysregulated activation of Hh/GLI1 signaling was well-known to impact on the multifarious signaling cascades of many human malignancies: the Smo- or GLI1-mediated activation of Hh signaling promotes the phosphorylation of MDM2 and the ubiquitination and degradation of p53, leading to the inhibition of p53-medaited apoptosis of DNA-damaged cells [19]; the over-expression of GLI1 and GLI2 increased the self-renewal of the mammary stem cell through the up-regulation of BMI-1 [20]. Furthermore, in a transgenic mouse model, the conditional expression of GLI1 was revealed to induce mammary tumors [4]. These reports suggest a crucial role of GLI1 in the development and progression of human malignancies [2]. However, the molecular mechanism to activate the Hh/GLI1 signaling during mammary tumorigenesis has not fully understood. Based on the evidences from the present study, we propose the molecular mechanism of GLI1 activation as follows. In normal duct epithelium, GLI1 accumulates but is confined in the nucleus as part of a transcriptional repressor complex including TSHZ2 and CtBP. During tumorigenesis, TSHZ2 is down-regulated and then GLI1 is released from the complex, whereupon it exhibits the transcriptional activity and shuttles between the nucleus and cytoplasm. Target genes of GLI1 are induced, leading to contribute the cellular properties, such as the proliferation (by AEBP1) and metastasis (by CXCR4) of breast cancer cells [16, 21, 22] (Figure 6D). In this scenario, TSHZ2 down-regulation functions as a “non-canonical” activator of Hh/GLI1 signaling cascade. Intriguingly, a previous report revealed that the promoter region of T*SHZ2* is not methylated in breast cancer cell lines [13], leaving a question of how TSHZ2 is down-regulated in breast cancer cells for future investigation.

We found in the present study that TSHZ2, but not TSHZ1 and TSHZ3, binds to GLI1 and GLI2. TSHZ family consists of three members, TSHZ1, TSHZ2 and TSHZ3, and to date, the knowledge about the function of TSHZ family members is limited: exceptionally, TSHZ3 was reported to form a ternary complex with SOX9 and Myocardin to disrupt the Myocardin-dependent expression of smooth muscle specific genes [23] and also to suppress a myogenic gene regulation by the association with Brg1-associated factor 57 (BAF57/SMARCE1), a member of ATP-dependent chromatin remodeling SWI/SNF complex [24]. TSHZ family members share the conserved zinc-finger domains and HOX domain, therefore, we speculate that TSHZ2 might bind to not only GLI1/GLI2 but also other transcriptional regulators that control the gene expression crucial for mammary tumorigenesis: TSHZ2 down-regulation-mediated derepression of these genes might contribute mammary tumorigenesis. For instance, we found that TSHZ2 over-expression in MCF-7 cells suppressed the expression of genes such as *ADAM28* [25, 26], which was reported to impact on the breast cancer biology (see GSE64351 for the details).

It has been thought that either the canonical or non-canonical Hh signaling pathway induces the expression of *GLI1* and contributes to the nuclear accumulation and transcriptional activity of GLI1 in cancer cells [1]. Indeed, pancreatic cancer cells, a representative type of cancer exhibiting the Hh signaling-dependent proliferation, express GLI1 predominantly in the cytoplasm, whereas the GLI1 expression is undetectable in normal pancreatic duct epithelium [27, 28]. By contrast, our study revealed that normal mammary duct epithelium expressed GLI1 predominantly in the nucleus, as previously reported [3]. At present, we do not know the biological role(s) of the GLI1 and GLI2 expression in the mammary duct epithelium; one possibility is that the expression of AEBP1, following GLI1 activation by TSHZ2 down-regulation, is prepared for the lactation, which requires the expression of AEBP1 [29].

In sum, we identified down-regulation of TSHZ2 as a key for the release of GLI1 from a CtBP-containing corepressor complex to lead the activated expression of GLI1 target genes during mammary tumorigenesis, and we speculate that in addition to GLI1 TSHZ2 might control other transcriptional regulators, which highlight the role of TSHZ2 in the development and progression of breast cancer.

## MATERIALS AND METHODS

### Cells and cDNA

Human breast cancer cell lines were obtained from the ATCC. Normal human mammary gland duct epithelium (HMEC) was purchased from LONZA. Immortalized normal human mammary gland duct epithelium (HMEC4*htertshp16*) was kindly gifted from Dr. Tohru Kiyono (the National Cancer Center Research Institute, Japan) and Dr. Denis Galloway (Fred Hutchinson Cancer Research Center). cDNA samples generated from breast cancer tissues and corresponding normal mammary tissues of two independent cases were purchased from BioChain.

### Microarray analysis

Total RNA was purified from cells for cDNA microarray analysis using Agilent 4×44K cDNA microarrays (Agilent Technologies) as previously described [27]. The microarray data are available online via the Gene Expression Omnibus (GEO) under the accession numbers GSE64350 [16] and GSE64351.

### Plasmid and lentiviral vectors

Expression vectors were constructed as follows: myc-tagged human TSHZ1, TSHZ2, TSHZ3 and mutated or deleted TSHZ2 from pCMV-3Tag (Agilent Technologies); DsRed-tagged human TSHZ2 from pDsRed-monomer (Clontech); GAL4-fused TSHZ2 from pBIND (Promega); HA-tagged or FLAG-EGFP-tagged human GLI1 and GLI2 and FLAG-tagged human CtBP2 from pCMVTNT (Promega); and EGFP-tagged GLI1 and GLI2 from pEGFP-C (Clontech). Mutated and deleted TSHZ2 constructs were generated via PCR-amplification. Luciferase reporter vectors were constructed as follows: pG5TKluc from UAS-containing pG5luc (Promega) modified by the introduction of the thymidine kinase minimal promoter (TKmini); wtGBSx4TKLuc and mutGBSx4TKLuc from the luciferase reporter pGL3 (Promega) modified by the introduction of four copies of the wild-type or mutated GLI-binding site of the human *CXCR4* promoter. Lentiviral vectors expressing either human TSHZ2, GLI1 or LacZ were constructed from the CSII-CMV-MCS-IRES2-Bsd plasmid, which was kindly provided by Dr. Hiroyuki Miyoshi (RIKEN BioResource Center, Japan).

### Luciferase reporter assay and qRT-PCR

Luciferase reporter assays were conducted using the Dual-Glo luciferase system co-transfected with a control *Renilla* luciferase expression vector (Promega) as previously reported [7]. Quantitative RT-PCR (qRT-PCR) was performed using a StepOnePlus real-time PCR system (Applied Biosystems) in conjunction with probes for TaqMan Gene Expression Assays (Applied Biosystems) according to the manufacturer's protocol.

### siRNAs, antibodies and immunoprecipitation

The following 21-nucleotide duplex siRNAs against human TSHZ2 (siTSHZ2-1, siTSHZ2-2), as well as a control (siControl), were synthesized: siTSHZ2-1, 5′-caauuugguaaugaaguaudTdT-3′ and 5′-auacuucauuaccaaauugdTdT-3′; siTSHZ2-2, 5′-guagaagaauuauuaaguudTdT-3′ and 5′-aacuuaauaauucuucuacdTdT-3′; and siControl, 5′-acacauuacaucuauguaadTdT-3′ and 5′-uuacauagauguaaugugudTdT-3′. The following antibodies were used: anti-TSHZ2 antibody (LS Bioscience), anti-GLI1 (Novus), anti-CtBP2 (BD Biosciences), anti-CXCR4 (BioLegend), anti-AEBP1 (LSBio), anti-GAPDH (Santa Cruz Biotechnology), anti-myc tag, anti-HA tag (Cell Signaling), and anti-DYKDDDDK (FLAG) tag (Transgenic, Japan). Immunoprecipitation was performed as previously reported [30].

### Immunohistochemistry and *in situ* proximity ligation assay

From the archives of the Department of Pathology at Aichi Medical University Hospital, 36 mammary tissues harboring invasive ductal carcinoma of no special type (20 cases of estrogen receptor (ER)-positive cancer and 16 cases of ER-negative cancer) along with 18 normal tissues were selected for the study based on the availability of tissue samples. The use of the samples was approved by the Institutional Ethical Review Board. Serial sections from formalin-fixed, paraffin-embedded tissue samples were subjected to immunohistochemical staining. The immunoreactivity was semiquantitatively scored with a three-tiered scale (negative, 0; weak, 1; moderate, 2; strong, 3) and compared using a Mann-Whitney *U* test, as previously reported [28]. The correlations between TSHZ2 and AEBP1/CXCR4/GLI1 expressions were examined by Spearman's correlation coefficient. The subcellular distribution of GLI1 was estimated as one of three patterns: the nuclear dominant pattern, the cytoplasmic dominant pattern or both of nuclear and cytoplasmic distribution. The correlation between TSHZ2 expression and GLI1 distribution was examined by Fisher's exact probability test. SPSS software was used for the statistical analyses. For *in situ* proximity ligation assays, 2 μm tissue sections were cut from formalin-fixed, paraffin-embedded blocks of human mammary glands and were treated with the Duolink *In situ* PLA kit (Sigma) according to the manufacturer's protocol, as previously reported [31].

## SUPPLEMENTARY MATERIAL FIGURES AND TABLE


