# Supplementary material for: Down-regulation of the zinc-finger homeobox protein TSHZ2 releases GLI1 from the nuclear repressor complex to restore its transcriptional activity during mammary tumorigenesis
Source: Oncotarget. 2015 Dec 29;7(5):5690–701. doi: 10.18632/oncotarget.6788 (PMC4868714; doi:10.18632/oncotarget.6788)
Supplement: Supplementary file 1 [file oncotarget-07-5690-s001.pdf]

Down-regulation of the zinc-finger homeobox protein TSHZ2 releases GLI1 from the nuclear repressor complex to restore its transcriptional activity during mammary tumorigenesis

Supplementary Material

Datasets		Top 5% genes Up / Down		
Ma *et al.*	DCIS epithelium vs Normal	956 / 956	457 / 530	↑ 3 genes<br>↓ 5 genes
	Invasive ductal breast carcinoma vs Normal	956 / 956		
Radvanyl *et al.*	DCIS vs Normal	665 / 665	107 / 66	
	Invasive ductal breast carcinoma vs Normal	813 / 813		
	Invasive lobular breast carcinoma vs Normal	795 / 795		
TCGA	Invasive ductal breast carcinoma vs Normal	1019 / 1019	720 / 730	
	Invasive breast carcinoma vs Normal	1019 / 1019		

Supplementary Figure S1. Detailed information of dataset comparison.
We initially compared top 5% genes differentially expressed between normal mammary glands and breast cancers and then extracted three up-regulated (*ATIC*, *C1orf43*, *RAG1AP1*) and five down-regulated (*AMOTL1*, *CRYAB*, *FAM189A2, SDPR* and *TSHZ2*) genes as genes commonly changed between all datasets.

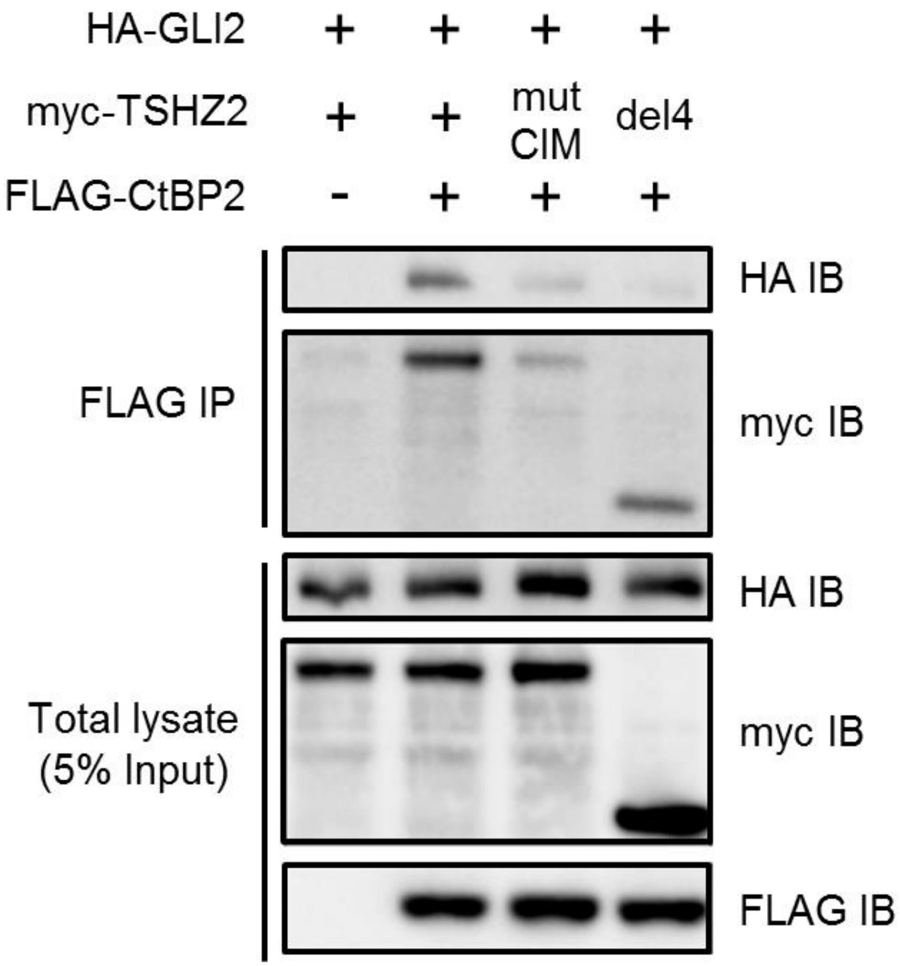

Supplementary Figure S2. GLI2 forms a ternary complex with CtBP2 in the presence of TSHZ2. HEK293T cells were transiently transfected with indicated expression vectors and served for immunoprecipitation analysis.

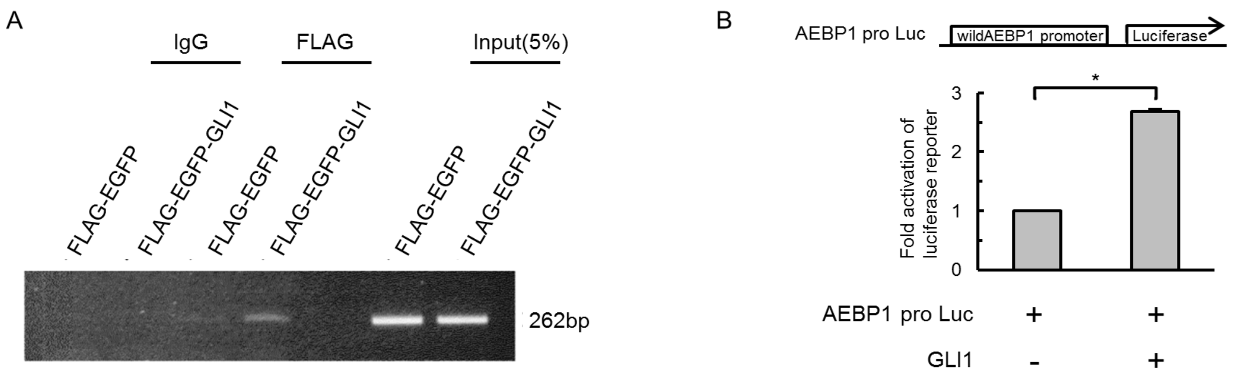

Supplementary Figure S3. GLI1 directly up-regulates the promoter activity of human *AEBP1* gene.
A, Chromatin immunoprecipitation (ChIP) assay. Cells were transiently transfected with either FLAG-EGFP-tagged GLI1 expression vector or a control FLAG-EGFP vector. After a brief cross-linking with formaldehyde and sonication, the cell lysate was subjected to co-precipitation with Dynabeads M-280 conjugated with either anti-FLAG antibody clone M2 (SIGMA) or an isotype-matched control IgG. PCR primers for *AEBP1* gene were designed to amplify the fragments containing the GBS sequence as follows: 5'-CCGCTCGAGGCTATCCGCGCGGGAGTG-3' and 5'-CCCAAGCTTCAGGGGCTCTGGGTCTCTGGGAAAG-3' (underline, matched sequence with human *AEBP1* gene). B, lucirease reporter assay. The promoter region of human *AEBP1* gene (1292bp) was PCR-amplified and cloned into the firefly luciferase reporter vector pGL4-basic (Promega). Cells were transiently transfected with either FLAG-GLI1 or a mock along with the reporter vector (AEBP1-Luc) and a control *Renilla* luciferase expression vector (Promega). The luciferase reporter assays were conducted using the Dual-Glo luciferase system. *Columns*, means of three independent experiments; *bars*, SD. *P<0.01.

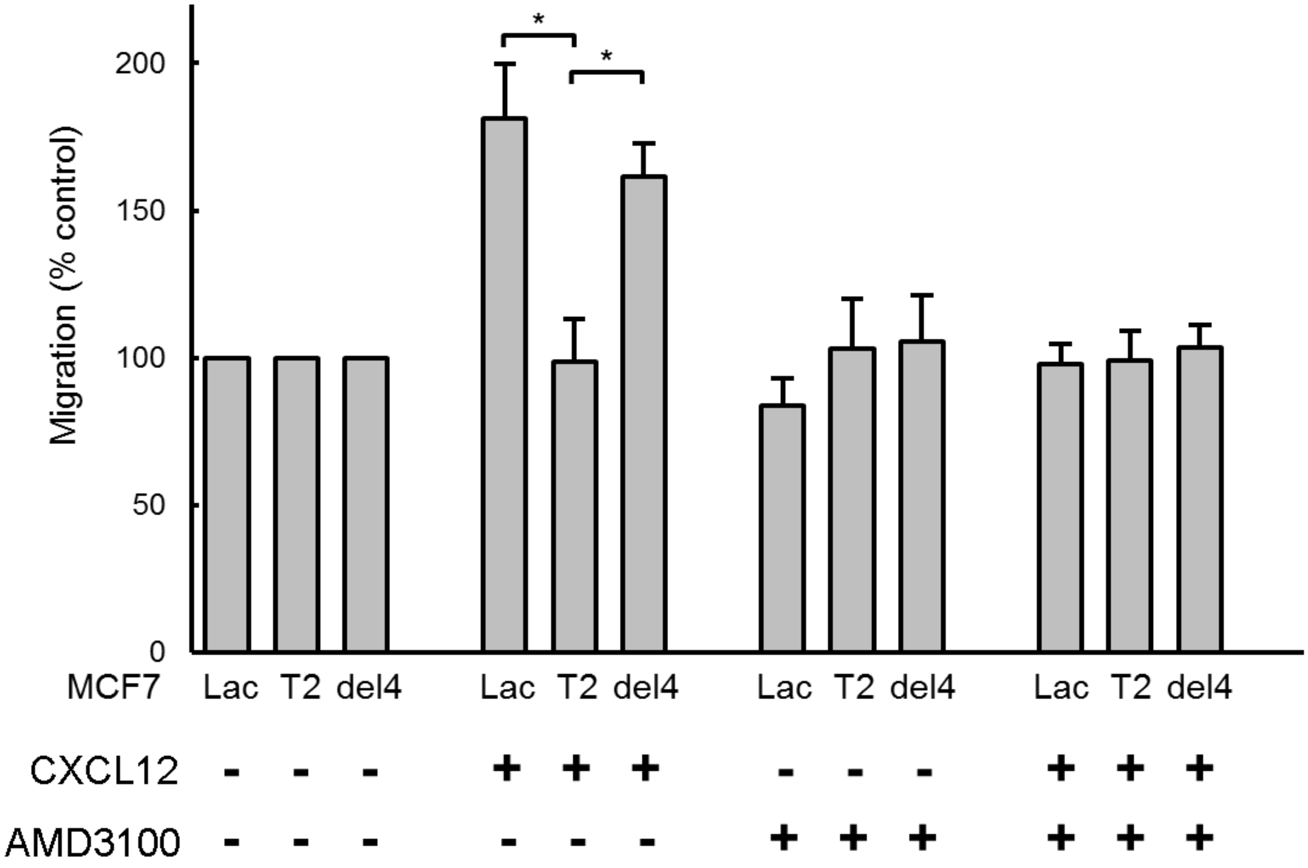

Supplementary Figure S4: TSHZ2 suppressed the CXCL12-induced MCF-7 migration.

Cells pretreated with either AMD3100 (100nM; SIGMA, USA) or a vehicle (DMSO) were resuspended in DMEM containing 5% bovine serum albumin and applied into the top chamber. CXCL12 (200ng/ml; R&Dsystems, USA) was applied as an attractant in the bottom chamber. bars, SD; *, $P < 0.01$.

Lac, LacZ; T2, TSHZ2; del4, $TSHZ2^{del4}$

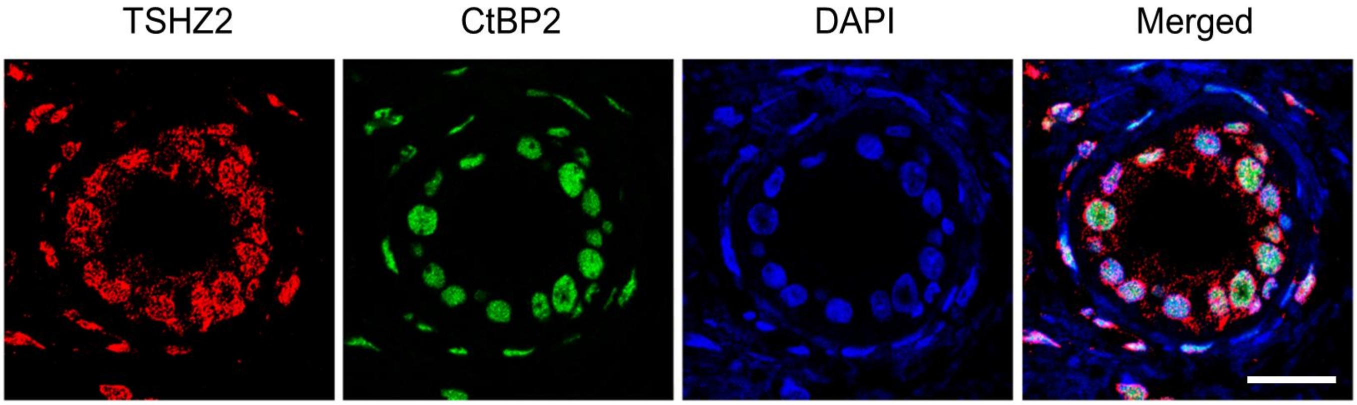

Supplementary Figure S5: Immunofluorescence staiing of normal mammary duct.

The section from formalin-fixed, paraffin-embedded tissue samples was subjected to immunofluorescence staining. Secondary antibodies conjugated with Alexa Fluor 555 (for TSHZ2) and Alexa Fluor 488 (for CtBP2) were used. Note that TSHZ2 and CtBP2 were co-localized in the nucleus. *Bar*, 20μM

Supplementary Table Case lists for immunohistochemical analysis.

Normal breast

Case No.	Intensity					GLI1
	TSHZ2	GLI1	CXCR4	AEBP1	CtBP2	localization
1	3	1	0	1	1	NC
2	3	1	1	1	1	N
3	3	1	1	1	1	N
4	3	1	1	1	1	N
5	3	1	0	1	1	N
6	3	1	1	1	1	N
7	3	1	1	1	1	N
8	3	1	1	1	1	N
9	3	1	0	1	1	N
10	2	1	1	1	2	N
11	3	2	0	1	1	N
12	3	1	1	1	1	NC
13	3	1	0	1	1	N
14	3	1	0	1	1	N
15	3	1	1	1	1	N
16	3	1	1	2	1	N
17	3	1	1	1	1	N
18	3	1	1	1	1	N

Invasive carcinoma

Case No.	ER status	Intensity TSHZ2	GLI1	CXCR4	AEBP1	CtBP2	GLI1 localization
1		2	2	2	2	2	N
2		1	2	2	1	2	NC
3		2	1	3	1	3	N
4		2	1	2	2	2	NC
5		2	2	2	1	2	NC
6		1	2	3	1	2	N
7		2	2	2	1	2	N
8		1	2	1	1	1	NC
9		2	2	3	2	3	N
10	ER(+)	2	2	2	1	2	NC
11		2	2	2	2	1	N
12		1	2	3	2	1	NC
13		2	2	2	1	2	NC
14		1	3	2	1	2	C
15		1	3	2	2	2	C
16		1	2	3	1	3	NC
17		1	3	3	2	1	C
18		2	2	2	2	0	N
19		2	2	3	1	1	NC
20		1	2	3	2	3	NC
21		1	3	2	3	1	C
22		0	3	3	1	2	C
23		0	2	2	2	2	NC

24		0	2	3	2	2	NC
25		0	2	2	3	2	NC
26		0	3	3	2	2	C
27		0	3	3	2	2	C
28	ER(-)	0	3	3	3	3	C
29		1	3	3	1	3	C
30		0	3	2	2	3	C
31		1	3	2	2	3	C
32		1	2	3	2	1	C
33		0	3	3	2	1	C
34		0	3	3	3	2	C
35		1	2	3	1	3	NC
36		0	2	2	2	3	NC
